# Analysis of an Important Work, Published a Few Years Since in Denmark, and Hitherto Unknown in England, on the Leprosy of the West in the Middle Ages, with a Supplement Relating to the History and Knowledge of the Disease

**Published:** 1803-06-01

**Authors:** Phil. Gab. Hensler

**Affiliations:** First Physician to the King of Denmark, and Professor of Physic at Kiel


ANALYSIS *of an important* WORK, *published a few Years since in* DENMARK, *and hitherto unknown in* ENGLAND, *on the* LEPROSY *of the* WEST *in the* MIDDLE AGES, *with a* SUPPLEMENT *relating to the* HISTORY *and* KNOWLEDGE *of the* DISEASE; *by* PHIL. GAB. HENSLER, *First Physician to the King of Denmark, and Professor of Physic at Kiel. Communicated to the Editors.*

THIS work will be a durable monument of the industry, judgment, and erudition of its author. No one can properly appreciate the great difficulties Prof. H. had to encounter in his undertaking, who has not experienced the obscurity in which the medical history of the middle ages is involved, and what profound skill in the barbarous language of those times is required to obtain a thorough knowledge of a disease, but few traces of which remain. A writer, who would seem to have studied nature, not books; who, amply furnished with literary treasures, has reaped an abundant harvest from extensive reading; and who, at the same time, possesses great acuteness of intellect with profound judgment, has overcome all these difficulties in such a manner, that the voice of the public cannot fail to bestow on his labours the loudest applause. It is not enough to say, that this work, which contains a complete history of the Leprosy, is so perfect in every respect, as far to excel every thing before written on the subject; but it appears to us as the best introduction to the history of our art, such as it was in the middle ages. In his history of the Lues Venerea, our author showed his opinion of the manner in which a history of the art of physic should be written; and had the writers of the present day pursued the path he there pointed out, the art would unquestionably have derived great benefit from their labours. This mode he has followed in his history history of the Leprosy, adverting to those circumstances that relate to the general history of the art. These, however, he touches briefly, and without going out of his way, making them accessaries only, not his principal objects; but they appear of the more moment, as they are eminently the fruits of judicious study.

The professor candidly owns, that his researches into the history of Syphilis, which he compiled a few years ago, afforded him considerable information respecting the Leprosy; for in the middle ages the theory of this disease was much more elaborate, and the rude physicians of those days were more diligent in fashioning their hypotheses into systems, to guide them in this unknown land. The humoral pathology too opened their eyes to the observation of some external varieties of this disease.

In the first chapter the author investigates the traces of the Leprosy to be found in writers both of the east and west. From the east, from Phœnicia and Egypt, where the disease is endemial, he proceeds to the west. As early as the ninth century, hospitals for lepers were every where to be found. The Arabian writers, who describe the leprosy, are briefly enumerated. Johannes Serapion and Janus Damascenus are the same person. The writings of Rhazes display little learning. (The learned author does not read Elchani.) No one of the Arabians is superior to Haly Abbas. (We think Rhazes.) Ebn Sina wrote as a philosopher. (We cannot judge properly of the doctrines and writings of the Arabians, till we have been made acquainted with their schools by physicians skilled in the Arabic language.) Constantine Africanus was the author of the system, to which subsequent writers adapted the theory of four species of leprosy. (Was not the whole of this theory invented by Ebn Sina, or borrowed with a little alteration from the Greeks themselves, from Paulus and Ætius, and adopted all by the Arabians?) Roge and Roland approved castration as the grand remedy. (A passage in Aretæus misunderstood, where he says, that the genitals have fallen off without injury in elephantiasis, gave rise to this error, which we know was afterward applied to the cure of the lues venera itself.) Theodoric was not a bishop, but a medical practitioner in Catalonia: he first gave a better description of this horrible disease than his predecessors. Lanfranc's, however, is deemed still preferable. Gilbert, of England, who was already acquainted with the spiritus Mindereri, is commended by our author.

 It

It seems altogether astonishing, that Theodoric of Spain, Gordonius of France, Salicetus of Italy, and Gilbert of England, should have given perfectly accordant histories of this multiform disease nearly in the same age. We cannot admit in its full extent the two periods established by our author in the barbarous ages of latinity, though they have some appearance of truth. That the Emperor Frederic II. was the first who promoted the study of the Greek physcians, and that the number of those who blindly followed the Arabians in consequence diminished, we do not deny; but we cannot grant, that the monocracy of the ancient Arabians was destroyed by his endeavours. There is unquestionably a striking difference between the barbarous Latin writers of the fourteenth century and those of the twelfth. It seems, however, wholly referrible to the reading of the latter physicians, which was assisted by better translations made directly from the Greek. But the Greeks were read before the time of Frederic II. though in translations executed by Arabians. Haller is by no means to be credited, when he says, that barbarous latinity reached its acme at the beginning of the fifteenth century. The history of the leprosy is brought down to Paracelsus and John Gersdorf.

A more recent case of a true elephantiasis, that followed a veneral infection, is added.

In the ſecond chapter the leproſy is deſcribed, ſuch as it appeared in the weſt during the middle ages. We cannot admit with our author, that the hypotheſis of four primary humours, on which the theory of all diſeaſes reſted above a thouſand years, was already eſtabliſhed in the writings of Hippocrates. Whence indeed could Hippocrates have derived this theory? The philoſophy of Heraclitus, which he followed, contained nothing like it: nay, the humoral pathology would have contradicted the reſt of the fragments of the theory of Hippocrates, περι ενορμωντος, περι εμφυτου θερμου, &c. This theory might be much more clearly derived from the philoſophical writings of Plato, to which it is clear the earlier followers of Hippocrates adhered. Thus the humoral pathology entered into the υποβολιμαια of Hippocrates. Praxagoras of Cos was the firſt, as far as we know, who brought forward this doctrine in its full extent.

Our author excellently delineates the nature of this Proteus malady, which ought rather to be conſidered as an univerſal diſeaſe than a particular ſpecies. In the weſt the following evident concomitants of this diſeaſe were obſerved.

*Morphea*

*Morphea alba*, the firſt ſpecies of *vitiligo*, the *ἀλφος** of the antient Greeks, *al wazehh*† of the Arabians, the *bohak*‡ of Moſes, was a ſpot of a milky whiteneſs, (this the Arabic word likewiſe indicates, as it alſo ſignifies milk,) inſenſible, and without any change of the ſkin beneath. In conſequence of this inſenſibility acupuncture was employed, to diſtinguiſh a harmleſs ſpot from the leprous. But if it aſſumed the form of lentils, it was called by the Greeks *φακος*, *φακιος*; by the Romans *lentigo*; (by the Arabs *baraſch* ||). If the author believes that the *ſeeth* of Moſes ſignifies this *lentigo*, he is greatly miſtaken.

*Morphea nigra*, of a brown colour, was another ſign preceding the confirmed diſeaſe, approaching more to the *impetigines*. *Vitiligo μελας* of Celſus; *αλφος μελας* of Paulus; the *εφελις* of ſome of the antient writers. (*Al khalaf* of the Arabians. The root خلف, inclining to black. *Al guaſem*, or rather *al waſchem*, was a very different ſymptom of the leproſy, though our author claſſes it here.) It irritates the ſkin, vehemently excites itching, and a deſquamation of the epidermis follows.

*Impetigines & ſerpigines variæ* precede the proper eruption of the leproſy. They are called *impetigines*, if they remain in the ſame place; *ſerpigines*, if they ſpread from one place to another. To theſe are to be referred the λειχηνες αγριαι, ψωραι, λεπραι, κνησμοι, of the antient Greeks; the *papulæ feræ*, *pruritus*, *pruriginoſæ papulæ*, *porrigines*, &c. of the Romans; the בהרת of Moſes; the *waſchem* of the Arabs, (called *al guaſem* by Henſler; *goſſone* by Salicetus,) from the root وشم, *ſtigmatibus pinxit cutem*, which the iſlanders of the Pacific Ocean call tattooing.

Though theſe *impetigines* have a red colour, they were antiently called φακωδες υπερυθρον, *ſemion* by Celſus; *miſpach* by Moſes, from the ſame root as the *ſafathah* of the Arabians, (سفح, *effudit*, as if the redneſs were poured out), by whom it was alſo called *dem almit* (dead blood), *gutta roſacea*, *aura ceruina*, (from *auerum*, a beaſt of burden, and *ceruina*, a wine cellar,) as if it were an animal employed in carrying red wine. It

† Not al guada, as our author writes it. The root is وضح ſplenduit, as if it were a ſhining mark.

‡ From the Syric root ܒܗܩ, fulſit, ſplenduit.

|| The author is miſtaken in his opinion, that this diſeaſe was called puſtules by the Arabs. Baraſch (Ebn Sina, lib. iv, fen. vii, tr. 3, c. 6.) ſignifies a ſpot in the ſhape of a lentil. The root is uſed of particoloured horſes, ſuch as we call dapple-grey. Baraſch (ش) muſt not be confounded with baras (برص), of which notice will be taken further on.

It was alfo called *buzicagua* (from *buza*, a wine-veffel, and *cahua*, wine of Cahors). Perhaps this was the *mal de rofa* of Afturia.

If the impetigines were of a brown colour, they were called αλφοι μελανες by the Greeks; *faphachath* by Mofes; *bohak al afud* by the Arabians, and came nearer to the *morphea nigra* mentioned above. Another difeafe follows as a precurfor of leprofy, which puts on various appearances, and comes under the names of *furfures*, *alopecia*, *ophiafis*. *Furfures*, επαναςασεις συκωδεες, ογκοι συκωδεες, πιτυριασεις, *tinea capitis*, with moift ulcerations, were frequently harmlefs. (Levit. xiii, 6, Mofes declares that הַמִּסְפַּחַת is innoxious. So does Ebn Sina, lib. vi, fen. vii, tr. i, c. 24. Hhafis fays, that حزاز* is not very dangerous.) But if the ulcers penetrate deeply, and if at the fame time a cancerous ichorous cacochymy be prefent, Mofes pronounces his *mispahhath* leprous. In the middle ages it was called *zerna ulcerofa*, *derbia*.

The ετεροχροια of the hair, attended with thinnefs of it, and falling off, was called *alopecia*. In the books of Mofes we have נֶתֶק†; in Ebn Sina, دا الثعلب *daalts' aleb* (*morbus vulpium*). Mofes properly diftinguifhes נֶתֶק (*alopecia*), from גַּבַּחַת (*baldnefs*), which Henfler does not appear to have obferved. Baldnefs commonly begins on the fore part of the head, *alopecia* on the hind part. Our author juftly conceives *tyria* to be the fame with *alopecia*, or rather with *ophiafis*; for it was a baldnefs attended with falling off of the hair, and eroding ulcerations in the neck. But there was another *tinea*, which was a confirmed leprous affection. (This *tyria*, of which we now fpeak, Ebn Sina exprefsly calls دا الحية; and he defcribes it fo diftinctly, as to leave no doubt of his meaning by it that harbinger of the leprofy, which later writers call *monile afturienfe* ‡.—Another fpecies of alopecia was called the *mentragra* of Pliny: this too, like all the other fymptoms juft enumerated, was inclined to degenerate into the leprofy itfelf.

Our author next returns to *maculæ*, *lentigines*, and *panni*; which however, after briefly confidering, he rightly refers to the *morpheæ* before mentioned. If he have not perfectly underftood the writings of the Arabians, here and in other places, it muft be afcribed to our verfions, made by Gerardus Car-

* The root حز, fcalpfit fe.

‡ *Le collier d'Afturie*, Thierry. The Afturians themfelves call it *gargantilla*.

monæńfis, monæenfis, Alpagus, and Rinius, which are corrupt, and altogether ufelefs. Perhaps therefore it may not be amifs, if we tranflate from a fplendid edition in Arabic, publifhed at Rome in 1593, a paffage, which our author has quoted in page 70, and from which, mifled by fome bad verfion, he frames an etymology and defcription not fufficiently accurate.

Ebn Sina, Lib. IV, Fen. VII, Tr. 2, c. 6.

'*De fanguine mortuo, lentigine fufca, & morphea nigra.*'

'*Namasch** (ferpigo rubra, φακιον ὑπερυθρον) and *fanguis mortuus* take place, when the blood in a diffolved ftate dilates the orifices of the veins, fo that the blood is feparated into craffamentum;† or when the acrimony of the blood produces διαπηδησιν; fo that, being retained fome time under the furface of the fkin, it becomes thick and atrabilious. But its (fuperficial) place is apparent from its figure and colour. If it incline to red, it is called *namafch (ferpigo rubra)*; if to brown, *barafch (lentigo fufca)*; and a fpot that is altogether dry, and void of moifture, is called *khalaf (morphea nigra)*. Some indeed call fpots in the fkin *khalaf*. Moft frequently it happens, that the lips of thofe who have *namafch* are chopped, from the too great drynefs of their temperament.'

If any one will take the trouble to compare this with the other known verfions, he will quickly perceive what numerous and important miftakes our tranflators commit. From the paffage juft given, what our author fays of lentigines, in page 75 and following, may be corrected. When he affirms, in p. 76, that the *lentigo rubra* was confidered as innocuous in the middle ages, though Hippocrates and Celfus declared it to be of a malignant nature; this is to be explained by the difference obferved between the precurfory fpots of the late writers, and the red appearances in a more advanced ftage of the difeafe.

Prof H. ingenioufly explains a difficult paffage in Hippocrates (Epidem. lib. iii, near the end), where the Coan fage fpeaks of thofe who are attacked by phthifis when an epidemic prevails. Το εἶδος *eorum notabant* το λειον, το ὑπολευκον, το φακωδες ὑπερυθρον, το χαροπον, λευκοφλεγματιαι, πτερυγωδεες, *areæ, maculæ nitentes, lentigines rubræ, oculi pulverulenti, humores frigidi, prominentia humerorum.* The fenfe of this paffage appears to be: they whofe bodies abound with a leprous humour are prone to phthifis.

---

* The root is نمش, *punctis variegatis confperfus fuit.*

† صربة is ufed of the coagulation of milk and of honey.

Our

Our author next proceeds to *puſtulæ*, which in the barbarous Latin are called by the common term of *ſahaphati*. What he ſays of this kind of little ulcers is not ſo eaſy to be explained, ſince the writers of the middle ages appear not to have underſtood their Ebn Sina themſelves. We conceive the *ſa'phah* of Ebn Sina to denote an ulcer of any kind. شير بنج, *ſhir-benadſch*, expreſſes an abſceſs, with a fat baſe, which the tranſlators call a *honeycomb*. باحثة, *babhith*, partakes of the confuſion that generally prevails in theſe words*. If, therefore, the writers of theſe barbarous ages uſed at a venture the words of Ebn Sina, which they did not underſtand, can we deduce any ſolid concluſions from them? Indeed, with reſpect to thoſe writers, our author juſtly aſſerts, that they adapted the various affections of the ſkin to the different diſorders of the humours. It was cuſtomary to aſcribe *morphea alba* to phlegm, *nigra* to melancholy, *lentigo* to choler, *puſtula* and *boſſor ulceroſum* to thick blood. Still the miſtakes we have juſt noticed, and the obſcurity in which the writings of the Arabians are involved, form no excuſe for thoſe who neglect them, and think ſlightly of the merits of others who purſue the laborious ſtudy. We ſhould not forget what Galen ſays: Τολμητεον τε και ζητητεον το αληθες, ει γαρ και μη τυχωμεν αυτȣ παντως· δηπȣ πλησιεςερον, η νυν εσμεν, αφιξομεσθα. We may hope too in this reſpect, πλησιεςερον αφιξεσθαι, η νυν εσμεν.

At length the profeſſor comes to *gutta roſacea*, or a ſpecies of *morphea rubra*, which he excellently deſcribes. Whether the *badeſchanem* of Ebn Sina (in our author, p. 89, *albedſamen*) ſhould be referred to this we cannot ſay†: but it is certain, that the ſame diſeaſe occurs in Paulus and Actuarius under the name of ὑπερυθροι ἐλεφαντες.

Next follows *lepra pſorica*; λεπρα of the Greeks; חָרָב of Job, from حرش, *ſcalpſit*. But of the Arabs دا لاسود, *morbus leoninus*. The *krimmiſche krankheit* (diſeaſe of the Crimea) of Pallas. In p. 91, the author profeſſes not to know what Rhazes and Ebn Sina mean by their *baraſſem*, which they reckon among *panni* and *lentigines*: if, however, he had

* Perhaps from باحم, *raucus*; becauſe exanthema was accompanied with catarrh and hoarſeneſs.

† We do not think it ſhould however; for the Perſians uſe the expreſſion باد شنام, *a ventis ortam ægritudinem, flatibus repletam puſtulam*. Perhaps *variola ſiliguoſa, alve repleta*.

read read the Arabian writers themfelves, he would foon have perceived, that what is called *baraffem* in bad tranflations, was no other than the *barafch*, or *lentigo variegata.* He rightly obferves, that Ebn Sina was the firft who called the fourth *impetigo* of Celfus *baras nigrum.* (For Rhazes and Haly Abbas do not call it *baras*, but *kuba* *: the old Arabians, who tranflated the Greek Phyficians into Arabic, always put قوبا, *kuba*, to exprefs λεπρα.) We may here mention, that the paffage quoted from Ebn Sina, in p. 14, is to be found, lib. iv, fen. vii, tr. 2, c. 9.—This *lepra pforica* is called likewife *zerna* or *malandria:* in Norway it occurs under the names of *fpedalfkhed* and *radefyge.* Our author thinks, that this malignant impetigo was of a fyphilitic nature; but this, we muft confefs, appears to us to want demonftration. We do not know, that buboes were not common to the leprofy as well as to the venereal difeafe; but we fay it with fubmiffion to the authority of Dr. H. who is eminently verfed in this matter. This leprofy was contagious; it was called in the middle ages St. Mævius's difeafe; and they who were affected by it were compelled to carry on a ftaff hands made of wool, that any perfon meeting them might be warned to avoid them.

The *lepra alba* however, *baras album* of the Arabians, λευκη of the Greeks, νεσος φοινικιη of Hippocrates, צָרַעַת † of Mofes, occafions confiderable confufion, fince neither the Greeks nor Arabians have diftinctly noted the differences between this and the preceding fpecies of confirmed leprofy. From *morphea alba* proceeds *lepra alba*, which was extremely prevalent among the jews, but not fo much in the weft. The critical effort of nature confifts in the expulfion of a thick morbific matter toward the furface of the body, which is greatly irritated by it. We cannot wonder, that neither the Arabians nor the barbarous Latin writers have given an accurate defcription of this difeafe and its progrefs; for it is their common fault, to join the definition of a difeafe immediately with its treatment, and to pay no attention to its hiftory.

Our author adds the treatment of the *lepra alba* from Ebn Sina, but in this he commits feveral miftakes from his being unacquainted with the Arabic. The chief of the laxative medicines among the Arabians was حبي النيل, *grana nil.* Prof. H. is miftaken, when he confiders this as a parafitic plant

* The root is قوب, *excorticata fuit cutis.*

† The root صرع, albus fuit.

ufed in dyeing, or perhaps a *cocculus*. (The *nil* of the Arabians was of two kinds: *grana nil* were faid to be the produce of a creeping plant, with blue bellfhaped trifid flowers. Unqueftionably therefore the feeds of the convolvulus fcammonia Lin.* شجرة النيل, *arbor nil*, of which Ebn Sina fpeaks copioufly, was the indigofera tinctoria Lin., not the ifatis, as Bauhine fuppofes.) The *electuarium anacardii*, which the Prof. enumerates among the laxatives of Avicenna, was chiefly ufed externally, on account of its great acrimony: for both Avicenna and Haly Abbas clearly and in exprefs words recommend this. The *zufera*, which our author fuppofes to have been a fpecies of rue, was nothing more than the ocimum bafilicum Lin†. The Arabians had recourfe likewife to phlebotomy and fcarifications, but only till the fkin was irritated by the eruptions. They injudicioufly dreaded this eruption, which was rather to be defired ‡. Preparations of quickfilver were ufed only externally as cauftics; and not to produce falivation, as in lepra pforica. A great number of cauftics was recommended againft this complaint, among which the oleum tartari per deliquium likewife figured with the weftern writers, however the application of cauteries, or *coctura*, fell wholly into difufe. Mofes was much wifer, who declared, that when the eruption took place the patient was cured. It was the practice of the Arabians and the barbarous ages, to prick the fkin with fharp-pointed inftruments: hence the *cofmetic art*, with which the fick concealed this difgraceful diforder, was always of great importance.

The defcription of the confirmed leprous difeafe, *elephantiafis*, *leontia*; *dfchoffam*§ of the Arabians, differs in authors, according to the various appearances the diforder affumed. The hiftory given by Aretæus appears to our author poetical, and, though diffufe, yet very near the truth. Gerfdorf's divifion of the fymptoms into thofe that perceptibly change the appearance of the body, thofe that depend on the efforts of nature, and thofe that relate to the fecretions, appears perfectly rational. That the writers of the middle ages have not left us fo full a hiftory

* Should it not be convolvulus nil, which bears a blue flower, and has trilobate leaves? Tr.

† This plant is faid to have a ftrong fmell, οσφρησις. Perhaps the name *fafar* (for fo it fhould be read) was formed from the Hebrew צפירה, *corona*, for the fame reafon as the Greeks called it βασιλικον.

‡ Abu'l Cafem however recommends the actual cautery as an excellent remedy in *baras album* and *dfchoffam*: de Chirurgia, lib. i, fect. 47, 49, p. 95, 97, ed. Oxon.

§ جذام, the root جذم, digiti ei truncati funt.

of of the confirmed difeafe from its commencement as they might have done, is owing to the ignorance of the people at large, who could not even forefee that fpecies of contagion, which was propagated by the intercourfe of the fexes. Accordingly a defcription of the leprous fever, which was its ufual beginning, follows. (It appears to us a fpecies of hectic.) Reddifh or faffron-coloured urine, turbid, with a fabulous fediment, was an evident fymptom. In this period too, gonorrhœa, ulcerations of the penis, condylomata marifcæ, and hæmorrhoids, fometimes ufed to appear.

Next follows a hiftory of the eruption itfelf, particularly of the various deformities, that are wont to occur in the face. This the reader will allow to be very accurate; but to enlarge upon it here would take up too much of our room. At an early period the voice was altered, and grew fqueaking *(graciliabatur)*: the patient was made to fing, that the hoarfenefs might appear. The continual alterations of heat and coldnefs, and habitual chillinefs, obliged the fick to ufe furs even in fummer. The examination of the blood deferves to be noticed: the ferum being poured off, falt was fprinkled on the craffamentum, on which it remained unchanged if the patient were leprous, but diffolved if he were in health. When the difeafe was farther advanced, the complexion became brown, and the face was puffed up as in tympany: the hair fell off, and if plucked out fomething flefhy adhered to the root. The eyelids fwelled and became hard, as if interfperfed with millet feeds. The chief fymptom (according to Ebn Rofchd) was a rotundity of the eyes, a ftern fixed look, a conftant epiphora, and frequently an inverfion of the eyelids themfelves. The nofe was affected with ulcerations, and turned outward; the lips fwelled, grew livid and black, and were deformed with fiffures, tumours, and condylomata: the breath became extremely offenfive: the whole body was covered with filthy blotches. (What our author, from the Latin verfion of Ebn Sina, calls *additio glandulofa*, p. 149, is in the Arabic text no other than *faphath*, which the tranflators improperly derived from the Hebrew, ספת, *addidit.*) Glandular and fcirrhous fwellings fupervene, with general leannefs, and particularly a prominence of the fhoulders, which Hippocrates calls πτερυγωδεα. A diftortion of the limbs, particularly of the fingers, deferves to be noticed; and laftly death, from a complete ceffation of all the fenfes and functions.

The *lepra nodofa* is much more accurately defcribed by the weftern phyficians, and appears to be depicted with more lively colours by them, than by the antient Greeks and Arabians. This is the elephantiafis, which, red at the commencement, paffes on to the greateft infenfibility and ftupor of all the parts, with with hard tumours and tubercles in all the limbs. This leprofy is called *quietior* by Ebn Sina, becaufe its eruption takes place later, and its progrefs is more flow. It had the fpecific name of *leonina*, if the body were covered more abundantly with ulcerations, if the rednefs at the beginning verged more toward yellow, if the blood were in a more diffolved ftate, and if its progrefs were more rapid.

The *lepra tyria* was obferved only in the weft: for the *tyria* of the eaftern Arabians (which has been fubftituted to the text by the tranflators) is nothing but the *ophiafis*, mentioned above. This leprofy was fuppofed to be of a phlegmatic nature. Our author efteems the defcription of Gilbertus Anglicanus as claffical. The precurfory fymptoms were palenefs of the face, a livid fwelling, morphea alba, and various blotches. A leucophlegmacy of the whole body with a fhining fcalinefs, and here and there glandular tubercles. The fkin peeling off many times in a year, like that of ferpents: the eyes moift and ftupid: the habit of body fqualid, weak, lax, ferous: the urine watery, with a greyifh fanies [*glaucofitas faniofa*] (Gordon). This lepra tyria our author confiders as the more mature ftage of *leuces*, or *baras album*, the ultimate progrefs of which is defcribed with little accuracy by the antients. Hence too it appears, why the *lepra tyria* does not occur by this name in the Greeks and Arabians; and why the *baras album* is not found in the weft, as this is comprifed in the *tyria* by the barbarous Latin writers.

*Lepra alopecia*, another variety which our author calls *rubra*, does not occur in the eaft. The falling off of the hair, whence it derives its name, does not conftitute the characteriftic mark of the difeafe. The face, inflamed with red ulcers, grows ftiff with fanies (Gaddefden): putridity prevails throughout the body: the urine is red and turbid: there are no fwellings. Our author juftly conceives, that perhaps fomething fcorbutic prevails in this; and he calls it the cradle of fcurvy.

The *lepra nodofa* appears chiefly to have affected only fome of the fmaller limbs: hence the *elephantia* of the feet and of the hands, different metaftafes of the fame difeafe, but which writers commonly refer to different heads. Hence Aph. vi, 34, it is juftly faid: ὁκοσοι φαλακροι, τουτεοισιν κιρσοι μεγαλοι ου γινονται. For varices in conjunction with elephantiafis of the feet appear to have conftituted the critical metaftafis of alopecia.

Our author adds obfervations hiftorical and pathological. 1. Of the oriental leprofy, of which Egypt feems to have been the native land. The difeafe of Job was certainly the *lepra pforica*, as prof. H. ably demonftrates. The leprofy of the Ifraelites was the *baras album*. 2. Of the leprofy of the Greeks and and Romans. The *lepra alba* occurred here and there in the iflands of the Archipelago: but the *nodofa*, and more efpecially the elephantiafis of the extremities, were fcarcely known to the Greeks a century or two before the Chriftian era. (Plutarch. Sympof. viii, 9.) The leprofy of the Romans before the time of Cicero was the *impetigo*. Celfus confounded and jumbled together all the fpecies and varieties of leprofy. After that time the *mentagra*, a direful leprous difeafe, broke out. Our author confeffes, that he could not difcover the *elephantia alba* in Pliny, which Raymond and even Gefner found there. (If we may give our opinion, it is, that Gefner concluded Pliny fpoke of the *lepra alba* from the paffage in his *Hift. Nat.* lib. xxviii, 12. For thefe *lentigines* and *lichenes*, which disfigure the fkin with a white colour, are mentioned: thefe fpots are reftored to the natural colour by affes' fuet, &c.)

Prof. H. fhews, by powerful arguments, that the leprofy, ftrictly fpeaking, was not communicated to the countries of the weft by means of the croifades; but had remained in them from the oldeft times of the Romans. But he does not deny, that the leprous lues raged with greater violence after the time of the holy wars, as is proved by the numerous *mifellaria*, or lazar-houfes, which Matthew Paris eftimates at 19000. He ably depicts the ftate of the wretched lepers, who were likewife called *mifelli*, lazars. It was the cuftom, however, to pay a particular refpect to the lazars, as if they were martyrs expiating family fins. (We cannot here but remember the kind of religious refpect paid to the *cretins*, as Ackermann teftifies.) The lepers appear likewife to have been dead in law: nay, public interments were inftituted. The abodes of lepers were called *ftellæ* and *cucurbitæ*.

Our author next proceeds to the decline of the leprofy in the weft. The fymptoms of *lepra nodofa* gradually vanifhed: impetigines and cutaneous affections became more common toward the end of the 15th century. Thus at length the leprous conftitution paffed into the fyphilitic. Thus lues venerea, ftrumæ, and elephas (according to Ballonius) have fomething of affinity, and συγγενες. He likewife quotes fome more modern obfervations on fporadic leprofy, and the progrefs of leprofy to America. In p. 243 he fays, he does not know whether the leprofy be found in the interior parts of Africa. But Bruce informs us, Travels, book v, chap. 1, that the *lepra pforica* is very common in Abyffinia. The difeafe differs completely, as he affirms, from the Syrian leprofy; though it likewife commonly produces metaftafes to the feet. Bruce denies, that the difeafe is contagious. For the *lentigo leprofa* or *morphea* of Aleppo, fee the Travels of Count de Ferriere-Sauvebœuf.

The

The pathological explanations, which our author adds, merit the higheft praife. The obfervation of a fpecies of *lepra tyria*, which came under his own infpection, particularly deferves notice. Of the eruptive fever: of the affections of the genitals, particularly priapifm and fatyriafis: of nervous affections.

The extracts added contain, befide paffages from the Arabian and barbarous Latin writers, Peyffonel on the Leprofy of Guadaloupe from the Philof. Tranfact. Deieanius's Epiftle on the Leprofy of the Eaft India Iflands, Buechner on the Leprofy of Norway, called *fpedalfkhed* by the Danes, *fpetalfkat* by the Swedes, &c.

*Liverpool, March* 5, 1803. B. D.

